# Hemodialysis—Nutritional Flaws in Diagnosis and Prescriptions. Could Amino Acid Losses Be the Sharpest “Sword of Damocles”?

**DOI:** 10.3390/nu12061773

**Published:** 2020-06-14

**Authors:** Piergiorgio Bolasco

**Affiliations:** 1 Nephrology Consultant, Sardinian Regional Public Health Institution, 09047 Selargius, Italy; piergiorgio.bolasco@atssardegna.it; Tel.: +39-333-2914-844; Fax: +39-070-609-3240; 2 Chronic Kidney Disease Treatment Group of the Italian Society of Nephrology, University Street, 11, 00185 Rome, Italy

**Keywords:** hemodialysis malnutrition, amino acid losses, protein-energy wasting

## Abstract

This review aims to highlight the strengths and weaknesses emerging from diagnostic evaluations and prescriptions in an intent to prevent progression over time of malnutrition and/or protein-energy wasting (PEW) in hemodialysis (HD) patients. In particular, indications of the most effective pathway to follow in diagnosing a state of malnutrition are provided based on a range of appropriate chemical-clinical, anthropometric and instrumental analyses and monitoring of the nutritional status of HD patients. Finally, based on the findings of recent studies, therapeutic options to be adopted for the purpose of preventing or slowing down malnutrition have been reviewed, with particular focus on protein-calorie intake, the role of oral and/or intravenous supplements and efficacy of some classes of amino acids. A new determining factor that may lead inexorably to PEW in hemodialysis patients is represented by severe amino acid loss during hemodialysis sessions, for which mandatory compensation should be introduced.

## 1. Introduction

Technological advances in the field and an increased clinical tolerability to treatment have heralded a significant rise in the age-specific rates of incidence and prevalence of hemodialysis (HD) treatment, regardless of the presence of a range of comorbid conditions [1]. In 2016, a mean age of dialysis patients of 62.4 years (prevalence 134 patients/per million population (pmp)) was recorded in Europe, with spikes in Israel and Portugal of 365 and approx. 332/pmp, respectively [2]. In 2017, 29% of all hemodialysis patients were aged 75 years or older (64% men, 36% women) with a five-year survival rate of 55% [3]. It is an acknowledged fact that an increase in age of HD patients produces a proportional rise in frailty, particularly related to nutritional disorders [4], seriously affecting patient survival and quality of life. Nutritional disorders are frequently manifested during the first stage of chronic kidney disease (CKD), classified according to the degree of Glomerular Filtration Rate (GFR, mL/min/1.73 m^2^) [5]. As a general rule, nutritional issues become more pronounced from stage CKD3-CKD4 (GFR: 29–60 mL/min/1.73 m^2^) [6,7]. In patients with CKD and ESRD, metabolic and regulatory disorders including acidosis [8], systemic inflammation [9] and hormonal dysregulation may be manifested [10]. These disorders are largely attributed to the onset of hypercatabolism and risk of negative nitrogen balance. Clinicians should not underestimate aspects related to an incorrect application of dietary restrictions imposed by a hypoproteic diet in the more advanced stages of CKD, (GFR < 29 mL/min/1.73 m^2^) [11], including increased energy expenditure [12]. The negative influence afforded by a series of comorbidities and concomitant medications should also be considered. End-stage kidney disease (ESKD) patients moreover are frequently affected by loss of appetite, anorexia and gastrointestinal complaints as a result of severe dysbiosis and associated significant imbalance of uremic microbiota [13], together with increased gut mucosal permeability to protein-bound uremic toxins [14]. This situation may deteriorate further when an ESKD patient starts hemodialysis treatment. In addition to the issues listed above, patients may also experience (a) hypercatabolic status during and up to several hours after HD [15]; (b) oxidative stress and chronic inflammation [16,17]; (c) progressive decrease in diuresis with loss of residual kidney function (RKF) caused by hemodialysis treatment applied in the lack of a tailored hemodialytic dose as recommended by precision medicine [18]; (d) insignificant depuration of protein-bound uremic toxins (PUBT) (particularly indoxyl-sulphate and p-cresol [19]), (e) loss of amino acids during hemodialysis (over 0.8 g/year) with progressive decrease of muscle mass; and (f) increased insulin resistance [20,21,22]. In the absence of therapeutic/nutritional countermeasures, protein-energy wasting will develop [23]. The aim of this paper is to highlight diagnosis and therapeutic procedures based on the most recent knowledge.

## 2. Monitoring of Nutritional Status in CKD Patients: Affected Parameters

Deterioration into a state of malnutrition in CKD and HD patients [24] up until onset of PEW [25], is linked to a series of key parameters, as illustrated in Table 1. Indeed, several interfering factors should be taken into consideration in the diagnosis of malnutrition and/or PEW: When calculating BMI, ethnic origin should be taken into account and when determining hyperhydration status, post-hemodialysis dry weight should be considered. Anthropometric measurements should be taken by a skilled nutritionist, and plicometry to estimate fat mass [26] and bioimpedance analysis [27,28] should be performed preferably by the same nutritionist. These biochemistry parameters are not pertinent in patients with significant proteinuria (over 3 g/day) or nephrotic syndrome, liver disease, or cholesterol-lowering drug consumption. Creatinine is strongly influenced by protein intake, particularly the last meal (lunch or dinner) consumed prior to HD, and by muscle mass (sedentary or active) and age. The most effective methods based on assessing protein intake based on protein catabolic rate (PCR) should be applied, particularly as dietary assessment or recall may yield ambiguous or inaccurate findings [29]. Whilst estimation of urea nitrogen appearance (UNA) remains a valid parameter [30] in the assessment of CKD patients, when using a urea kinetic model equilibrated normalized protein catabolic rate (eqPCR) in HD patients should only be estimated in the presence of steady-state metabolism. Determination of eqPCR should be avoided if the patient is suffering from an inflammatory and/or hypercatabolic condition [31].

### 2.1. Blood Chemistry Parameters

In hemodialysis patients, results obtained for a series of blood chemistry parameters are not deemed as reliable as when evaluated in other chronic conditions. Malnutrition may be indicated at levels of albuminemia <3.8 g/dL and pre-albumin <30 mg/dL. The latter, also referred to as Transthyretin (transport protein for thyroxine and retinol), is a rapid marker for malnutrition based on serial measurements obtained over several days, although levels of this protein are influenced by age, sex, CRP, IL-6, Charlson Score and visceral body fat [32,33]. Total cholesterol < 100 mg/dL and decreases in non-HDL-cholesterol and non-HDL/HDL cholesterol ratio (including triglyceride-rich lipoproteins) have been paradoxically associated with increased all-cause and cardiovascular mortality in patients undergoing incident hemodialysis [34]. However, numerous other indicators may also not constitute reliable indicators, as an example: (a) low serum transferrin, estimated by total iron-binding capacity (TIBC) is influenced by iron deficiency, inflammation, poor quality of life in patients on hemodialysis [35], (b) creatinine is heavily influenced by muscle mass volume, hemodialysis adequacy, residual renal clearance, hypercatabolism by dialysis, protein food intake prior to sampling (e.g., previous meal), particularly when blood is drawn following the longer interdialytic period or during afternoon HD sessions [36], (c) serum leptin is one of the parameters underlying the onset of anorexia in hemodialysis patients, but cannot be considered an important correlation factor due to significant association with inflammation [37,38], (d) metabolic acidosis, together with low caloric intake, elicits muscle proteolysis, reducing the sensitivity of cells to insulin, boosting the presence of molecules such as ghrelin and leptin that act on the central nervous system (CNS), which in turn increase resting energy expenditure [39], and (e) lymphocytopenia may represent a confounding factor due to the frequent presence in HD patients of a sub-chronical disease-causing a decrease in lymphocyte count, including primary immune deficiencies and immune deficiencies secondary to malnutrition or zinc deprivation, excess catabolism, immunosuppressive therapy, HIV infection, systemic lupus erythematosus, certain viral infections, lymphoma, renal insufficiency, and idiopathic CD4 lymphocytopenia [40].

### 2.2. Anthropometric Indicators

Anthropometric methods may be validated for use in HD patients, although alone are not sufficient and should be associated with the other parameters described. In addition to body mass index (BMI), lean muscle mass should also be taken into account, particularly when values of <25 kg/m^2^ are detected and reduction in average arm circumference is 10% below the 50th percentile of the reference population. Mid-arm circumference, triceps skin-fold thickness and mid-upper arm muscle circumference were measured. Waist circumference was measured above the iliac crest, at the level of the hips and on the most relevant part of the buttocks; these parameters also correlate with cardiovascular risk. In addition to these findings, plicometry was applied to measure thickness of the skin on the arm. Finally, the assessment of muscle strength by means of specific tests is fundamental, although strength may be significantly influenced by age, cognitive status, smoking, alcohol intake and sedatives [41,42,43,44]. Body weight and consequent BMI should always be assessed prior to dialysis sessions in view of the potential for hyperhydration frequently manifested in HD patients. Subjective global assessment (SGA) is a widely-used sensitive technique used to assess nutritional status and is better correlated with health-related physical and mental quality of life aspects in HD patients [45,46,47]. The so-called “BMI paradox”, i.e., the association of high BMI with inflammation observed in dialysis patients, should also be taken into account. Recent data suggest a cross-sectional association of high BMI or abdominal adiposity with inflammation produced through alteration of circulatory cytokines, sequestration of uremic toxin in adipose tissue, and endotoxin–lipoprotein interaction, coronary artery calcification myocardial injury, a more proatherogenic profile in terms of inflammatory markers and adipokine expression, lower body composition reserves, and lower physical ability and decreased survival [48,49]. To date, therapeutic results obtained with regard to “sarcopenic obesity” have been disappointing. Weight reduction obtained through conservative or metabolic (bariatric) management and weight loss during dialysis treatment should be avoided [50,51]. Maintenance of body weight by means of adequate nutrition and physical activity is mandatory in preventing weight loss or onset of sarcopenia; body composition assessment and functional testing (handgrip strength, gait speed) should be carried out. Structured exercise programs have been proven to increase muscle mass and functional outcomes. Furthermore, high BMI (> 30–35) is associated with higher risk of transplant complications. 

### 2.3. Bioelectrical Impedance Analysis

Bioelectrical impedance analysis (BIA) is a method used to measure body composition based on the rate at which an electrical current travel through the body [52]. Electrical impedance is based on reactance which measures body cell mass, and resistance which measures total body water (TBW), with these two parameters being applied to calculate the phase angle (Å). Based on BIA measurement, a number of body composition parameters can be estimated, including body cell mass (BCM), fat mass (FM), muscle mass MM), free fat mass (FFM), and total, intracellular and extracellular body water (TBW, IBW, EBW). BIA however generally tends to overestimate muscle mass, although Å appears to be a useful bioelectrical marker in predicting and monitoring nutritional status in HD patients [53,54]. This important issue should be addressed when using BIA for clinical and research purposes. To better validate BIA, the equations generated to facilitate estimation of muscle mass based on factors including age, sex, height, weight, and lack of one or more should be taken into account. Monitoring of patients’ hydration status and weight gain in the interdialytic interval is fundamental. BIA measurements should therefore be taken once dry weight or ideal weight is purported to have been reached, where possible at least 30 min after dialysis and following urination [55,56]. Indeed, the HD population studied displayed a worsening of intracellular dehydration, hypervolemia and cell mass wasting [22], all of which may potentially result in a reduction in muscle mass and strength. Furthermore, in overweight and obese HD patients, BIA-derived FFM, BCM and Å are significantly lower compared to normal-weight patients and BMI-matched controls. Finally, Å adjusted for excess fluid after HD, age, and gender, may constitute the most potent predictor of malnutrition and survival in hemodialysis patients [57].

### 2.4. Main Nutritional Score Evaluations

The majority of the above-described methods, however, are expensive, cumbersome and not suitable for use in routine follow-up in hemodialysis patients. The mini nutritional assessment (MNA) consists in both a shortened form six-item screening tool (MNA-SF) and a full-length 18-item scale (MNA-LF). The full-length tool comprises 18 items aimed at evaluating a range of aspects: BMI, weight loss, arm and calf circumferences; lifestyle, medication, mobility and presence of signs of depression or dementia; brief dietary assessment and subjective assessment. Using the MNA, three specific groups are identified: MNA < 17 indicating malnourished patients, MNA 17–23.5 patients at risk of malnutrition and ≥ 24 normal nutritional status [58]. A study conducted by Holvoet et al. indicated MNA-SF as an appropriate and feasible tool for use in identifying nutritional problems in dialysis patients [59]. However, previous studies [60,61] had cast doubt on the reliability of SGA, malnutrition inflammation score (MIS) and (MNA). When applied to elderly patients, these methods are subject to limitations related to aging (e.g., memory loss, poor compliance, disinformation, etc.). This review also addresses the growing concerns over bias in the estimation of nutritional intake and the possibility that differential bias moves with stratification variables of analytical interest. Inaccuracies arising from the dietary recall of HD patients have long been an acknowledged issue, thus advising against use of this method on dialysis wards due to the high potential for error in dietary assessments, particularly relating to protein intake and calculation of energy expenditure [62]. Conversely, protein intake may be assessed indirectly, particularly in patients on a thrice weekly hemodialysis regimen, by calculating the protein catabolic rate obtained from the percentage or logarithmic variation between pre- and post-dialysis urea nitrogen values. However, it is imperative that HD patients are evaluated in a metabolic steady state, excluding acute or chronic intercurrent pathologies and therapies which may add to dialysis-induced hypercatabolism, thus rendering calculations unreliable [63,64,65]. In the case of HD patients on a once or twice weekly hemodialysis regimen who maintain residual diuresis with minimal residual kidney function (RKF), a new and efficient mathematical calculation algorithm is available for practical application of urea kinetic modeling. This is a freely distributed and open-source JavaScript tool called Solute Solver, derived from 35 dialysis and anthropometric parameters which may be applied to evaluate the normalized protein catabolic rate (nPCR) [66]. Another difficult parameter to calculate is resting energy expenditure (REE), particularly as HD patients have a higher REE adjusted for muscle mass than healthy controls. Body composition should also be determined and hypercatabolism taken as a reliable indicator of malnutrition [67]. Furthermore, REE is not influenced by the degree of renal function, although may be elevated during subclinical inflammation typical of hemodialysis patients [68,69]. In restoring the patient to initial dry weight, several proposed equations fail to take into account body temperature elevation during routine HD [70] or energy required for vasoconstriction to prevent fall in blood pressure as a result of a reduced water volume following loss through ultrafiltration. It should be underlined how this phenomenon is amplified in acetate hemodialysis where energy is needed for the conversion of acetate into bicarbonate by the muscles [71,72]. Dual-energy X-ray absorptiometry (DXA), based on the signals provided by two energy sources to provide a three-compartment model of body composition, has become the gold standard test [73]. DXA is a reproducible and reliable technique used to measure fat mass in healthy people, as well as in HD patients. Unfortunately, this costly device, which is nonportable and relies on operator proficiency, cannot be used as a practical or accessible bedside tool. Edema or hyperhydration render DXA less reliable than BIA in the precise detection of different body compartments, in particular total body water (TBW), in chronic HD patients. It should, moreover, be highlighted how these parameters should be detected only in advanced CKD stages by expert doctors or nutritionists through dietary recall and, more recently, dietitian-led telehealth coaching intervention, by applying the Healthy Eating Index. Actual calorie intake and degree of compliance of interviewed patients is hard to establish [63,74]. An additional method of proven validity is represented by the calculation of urea nitrogen appearance (UNA) [75]. Selected patients with a good RKF undergoing once-weekly hemodialysis showing good compliance with low protein intake (0.6/g/kg/day) were assessed over a six-day interdialytic program [76,77] and UNA calculated; this method provides an indirect estimate of dietary intake based on urinary nitrogen output, fecal output and body area nitrogen. 

## 3. Nutritional and Therapeutic Interventions

### 3.1. Ideal Diet for Hemodialysis Patients

Advice for HD patients [78,79] has remained unchanged for many years and the diet still prescribed today for thrice weekly HD regimens envisages a protein intake of 1.2 g/kg/day, 30-35 kcal/kg/day, sodium intake < 3–5 g/day, phosphate intake < 1000–1200 mg/day and potassium intake 2000 mg/day. The correct intake of phosphate and potassium is difficult to establish as this is affected by dialysis adequacy, the quantity of phosphate and potassium binding and state of the uremic microbiota. The use of lanthanum carbonate as a phosphate binder leads to a decreasing microbial diversity and lower network complexity [79,80]. In an incremental/infrequent HD strategy [81], whilst the exact protein intake in a twice weekly hemodialysis has not yet been established, there is agreement that protein intake should be 0.6 g/kg/day (50% animal proteins) [82,83]. However, the choice of infrequent HD protects RKF through use of high-flux and biocompatible membranes, particularly hemodiafiltration, use of ultrapure dialysate, a low-protein diet, and careful monitoring of metabolism and blood pressure [84]. 

### 3.2. Replacing Amino Acid Losses by Hemodialysis

An important issue has recently emerged with regard to the loss of amino acids (AAs) in dialysate. Indeed, due to their low molecular weight, AAs are lost in industrial quantities over one year of thrice weekly hemodialysis, particularly when using methods such as hemodiafiltration and hemofiltration, in which additional convective losses occur due to ultrafiltration. Recently, a study group on AAs kinetics in extracorporeal methods showed annual losses > 800 g/year in thrice weekly hemodialysis patients with a consequent, significant loss of lean body and, in particular, muscle mass protein [20,22]. Considering the more contained loss of Total AAs (TAAs) manifested using high-efficiency hemodialysis with a surface dialyzer area of 1.8 m^2^ over a 240-min session, losses could be managed by varying dialytic strategy as shown in Table 2.

The most severe metabolic consequences likely result from loss of essential amino acids (EAAs) such as threonine, tryptophan and lysine and from Non-Essential amino acids (NEAAs) such as tyrosine, aspartic acid, serine, glutamic acid and glycine, resulting in the onset of hypercatabolism and threatening muscle mass loss. Loss-replacement nutritional supplements have been proposed, with keto analogues being used to replace amino acids lost during hemodialysis. Keto analogues are made up of calcium salts, leucine, isoleucine, phenylalanine, valine and Ca-hydroxy-methionine, L-lysine, L-threonine, L-histidine, and L-tyrosine (alfa-kappa, Ketosteril ^®^, Fresenius Kabi, Bad Homburg, Germany), although taken alone are not sufficient to replace amino acid losses in HD patients. Moreover, supplementation may result in excessive doses of nitrogen, with each tablet containing 337 mg of this element; i.e., calculated on the dose recommended for 70 kg body weight, each patient would consume approx. 470 mg/day nitrogen, also implicating a potential interference with calcium-phosphorus metabolism (such as hypercalcemia) due to a higher calcium intake, with each tablet containing 45 mg calcium; i.e., for 70 kg body weight, an intake of approx. 570 mg/day calcium. This type of product should only be used in advanced stages of chronic kidney disease (CKD4-CKD5) in patients adhering to a very low protein diet regimen (VLPD) [85,86]. In a paper by Bellizzi et al., nephrologists and nutritionists are warned to monitor for probable poor compliance, particularly if the low protein diet is associated with AA intake through keto-analogues. Indeed, in patients prescribed a higher number of tablets or sachets (8–12 per day), compliance with this schedule after six months was limited to 64.5% [87]. Moreover, keto-amino-acid analogues represent a considerably higher cost than other commercial products (over EUR 4000 per year), and treatment could be successfully replaced by a tailored, more efficient and less expensive amino acid supplement EUR 600–700 per year). Very few trials have been conducted to date to evaluate AA supplementation in hemodialysis patients; based on our previous experience [88], the daily administration of 5 grams of a combination of 6 EAA, 2 NEEA, 2 BCCAA, with no metabolic accelerators, plus vitamins B1 and B6 for three months, obtained highly promising results (Table 3).

The results of this study led us to recommend the administration of a new amino acid combination of 20 main amino acids (EAAs, NEAAs and BCAAs), vitamins and micronutrients tailored to the quantities and qualities of amino acids lost through dialysis at the end of a hemodialysis day (Amino-HD, Professional Dietetics, Milano, Italy) [89], with administration during the interdialytic interval of an amino acid solution containing 10 EAAs with mitochondrial metabolic accelerators such as malic and succinic acids, group B vitamins and a minimum calorie intake (Amino-Ther, Professional Dietetics, Milano, Italy). Neither combination contains nitrogen or calcium. These new amino acid mixtures may increase cellular oxygen uptake (an effect produced by nitric oxide NO mediated by PGC-1alpha), the main regulator of mitochondrial biogenesis, thus promoting biogenesis and mitochondrial function by activating catabolic processes of amino acids. These new combinations contain a carefully calibrated dose of the aromatic amino acids tyrosine, phenylalanine and tryptophan, which are converted into protein-bound uremic toxins (PBUTS). These observations were made by comparing the difference between AA levels in plasma from arterial blood of HD patients and healthy subjects [20]. This metabolic effect could slow down or prevent decline into malnutrition and/or protein-energy wasting in patients required to sustain years of treatment and avoid the use of amino acids in muscle mass to produce energy [90]. A post-hoc analysis [22] confirmed a severe loss of AAs during hemodialysis and/or hemodiafiltration (HDF), with detection of a marked loss of total AAs (5 g/session), corresponding to more than 65% of all AAs. Regarding individual AAs, glutamine displayed a consistent increase (+150%), whereas all other AAs decreased after 12 months of HD/HDF. Only a few AAs, such as proline, cysteine, and histidine maintained normal levels. The most severe metabolic consequences may result from losses of EAAs such as valine, leucine and histidine, and from NEAAs including proline, cysteine and glutamic acid, eliciting the onset of hypercatabolism threatening muscle mass loss. In our patients, dialysis losses, together with the effect of chronic uremia, resulted in a reduction of fundamental EAAs and NEAAs, which over 12 months progressively led to a deterioration of lean mass, leading towards sarcopenia. Therefore, the reintroduction of a correctly balanced and tailored AA supplementation in patients undergoing HD to prevent or halt the decline of hypercatabolism into cachexia, is recommended. 

### 3.3. Reduction of Hypercatabolism by Hemodialysis

Although in the majority of cases, dialysis sessions are well tolerated, in HD, onset of the compartmental imbalance is asymptomatic, rapid and violent. Intra-dialysis inflammation plays a fundamental role due to contact with dialysis membranes, even the least biocompatible, by means of which activation of a class of monocytes responsible for the release of cytokines is inevitable. Hypercatabolism in dialysis patients is related to intradialytic loss of amino acids as well as cytokine activation [91,92]; interleukin-6 plays a central role in regulating whole-body, muscle and hepatic protein turnover during hemodialysis. CD14 + CD16 + lymphocytes play a central role in the release of cytokines (IL-1 IL-6, TNF-α) [93]. Furthermore, at the end of the session, a post-dialysis rebound of numerous molecules occurs, the most widely studied of which is urea, largely due to the ease of detection. This rebound of uremic toxins is well known and is first manifested by the redistribution of molecules such as urea, phosphates and β2-microglobulins from several cellular and intracellular compartments and in plasma water. The magnitude of this compartment redistribution is directly related to the purifying dialytic intensity, resulting in consequent hypercatabolism and further energy expenditure. It is well known that a short standard hemodialysis treatment corresponds to a protein catabolic rate (PCR) > 1.4 g/kg/day, with this value corresponding to the daily protein intake required by the patient to compensate for the increase in PCR linked to dialysis hypercatabolism [94,95].

To conclude this chapter, it should be highlighted how the oxidative stress manifested in chronic kidney disease is exacerbated by HD treatments using any type of dialysis membrane, through triggering of platelet activation (release of reactive oxygen species, ROS), failure to use ultrapure dialysate (endotoxins cross the membrane from poor quality dialysis water), or use of an acetate buffer rather than bicarbonate (ROS release) [96]. It still remains a very difficult task to prevent hypercatabolism produced by hemodialysis, although satisfactory results may be obtained by using less biocompatible membranes or membranes that reduce the passage of contaminants from dialysis liquid. Lympho-monocyte activation occurs in the presence of all types of HD membranes, thus underlining the need to use optimum sterilization methods, ultra-pure or sterile dialysis liquid flow and the most biocompatible biochemical composition to the hemodialysis machine [97,98,99,100,101,102,103,104,105,106,107,108].

### 3.4. Replacement of Vitamin Losses

Dialysis patients frequently present with reduced levels of a broad range of vitamins [109]. Reports focused on vitamin losses present in the literature relate solely to a few studies from the 1980s, mainly because the majority of studies have concentrated on the loss of vitamin D in its various forms. The results of these studies are of scarce utility in providing a tailored personalized therapy with vitamin D (oral or intravenous). The dosage of other vitamins (vitamins C, A and E) for the treatment of renal osteodystrophy is highly complex, lengthy and expensive, involving the use of reverse-phase high performance liquid chromatography. No significant reductions have been observed during any extracorporeal therapeutic option in vitamin A, B1, and B12 [110]. The only loss reported was for vitamin C, particularly when using hemodiafiltration methods of 8–230 µg/session, resulting in a significant reduction of plasma levels from 1.87 µ/mL to 0.98 µg/mL [111]. Vitamins C and E are both characterized by anti-oxidative properties, with vitamin C acting as an enzyme cofactor and enhancing mobilization of the ferrous form of iron to transferrin, thus increasing bioavailability and avoiding limitation of administration to prevent secondary oxalosis [99,112]. As a general rule, HD patients do not manifest losses of vitamin B1, B12, C and folate, as these are replaced intravenous at the end of a HD session, or orally with cycles of approx. 15 administrations three times per year [113].

### 3.5. Other Intravenous Supplements

When faced with an evident state of malnutrition or PEW, intravenous nutritional and caloric support should be provided. Ideally, this should be administered throughout the entire duration of the extracorporeal session [114, 115,116]. Parenteral nutrition administration must provide an adequate calorie intake (approx. 1000 kcal) from lipids and albumin, and the administration of amino acids during the session preferably avoided as these remain in the circulation for several minutes [117] and are eliminated by diffusion and ultrafiltration diffusion through the dialysis membrane [118]. This often produces nausea during the dialysis session. Furthermore, it remains unclear whether significant advantages regarding baseline characteristics or nutritional status are registered following administrating of intravenous nutrition during HD treatment [119]. It may therefore be advisable to administer nutrition after the hemodialysis session. Intra-parenteral administration should continue over a period of four to six months in order to restore a positive metabolic balance even in severely malnourished patients [120]. However, the majority of infusions currently marketed contain amino acids. These solutions are lipid solutions in the form of binary, ternary mixtures with the presence of medium- or long-chain triglycerides at 10%, 20%, 30% (10 kcal/g) essential fatty acids, vegetable oils such as refined soybean and olive oil, fish oil containing Omega-3 and vitamin E to avoid rancidity of the lipid solution. Mandatory procedures for intra-parenteral, intra-session administration provide for post-dilution infusion by hemodiafiltration, as this method allows lipid nutrients to be directly administered intravenously without passing through the dialysis filter. The infusion rate of the lipid solution (1800 mOsmL/kg) can be readily calculated by 1:6 dilution with the dialysis infusion liquid, thus preventing throughout the hemodialysis session plasma hyper-osmolarity on the venous system, particularly at the arteriovenous fistula [121]. The high costs could potentially be recovered through a reduction in the morbidity and hospitalization of patients in whom undernourishment or PEW have been successfully prevented.

### 3.6. Preserving Gut Microbiota in a Uremic Milieu

For many years, the microbiota has been underestimated; however, it is now an acknowledged fact that in advanced uremic stages the microbiota is significantly affected. Chronic kidney disease is characterized by an accumulation of protein-bound uremic toxins (PBUTs) such as p-cresyl sulfate (pCS), p-cresyl glucuronide (pCG), indoxyl sulfate (IxS), and indole-3-acetic acid (IAA). Each of these uremic retention solutes exerts toxic effects, and several have been associated with worsening outcomes in CKD patients, in particular with cardiovascular morbidity and mortality. All four PBUTs originate from the intestinal microbial metabolism, mainly from the aromatic amino acids (AAAs) tyrosine, phenylalanine and tryptophan [122,123,124,125]. These outcomes stem from a state of dysbiosis in bacteriological equilibrium with pathobionts overcoming symbiotic germs and becoming deranged in the tight gastrointestinal junction barrier resulting in an increasingly toxic milieu such as PBUTs, [126] which is highly toxic on a cardiovascular level. This in turn may elicit cardiac damage and nephrotoxicity, endothelial damage and diffuse endothelial injury [127]. Effective, targeted therapies for HD patients have not yet been well defined, with the majority of research work aimed at decreasing the levels of PBUTs rather than specifically curing the uremic microbiota [128]. Some authors have recently suggested the addition of nuts and/or vegetables to the diet [129,130], whilst others have performed studies on the efficacy of a new intestinal charcoal adsorbent [131]. A recent review by Bao et al. describes the ability of different polyphenols, such as anthocyanin, catechin, chlorogenic acid, and resveratrol, to regulate intestinal microorganisms, inhibit pathogenic bacteria, and reduce inflammation [132]. Another study group conducted a randomized, placebo-controlled pilot study in HD patients to establish whether administration of a symbiotic, either individually or in association with divinylbenzene-polyvinylpyrrolidone (DVB-PVP) cartridge, could reduce the production of uremic toxins [133]. In view of the complexity in establishing full composition of the microbiota, therapeutic trials aimed at correcting uremic dysbiosis are few, unsatisfactory and inconclusive. Despite a series of attempts to date, no effective therapy using prebiotics, probiotics and symbiotics to maintain a healthy microbiota in hemodialysis has yet been defined [134,135]. However, tailored amino acid supplementation may produce a certain rebalancing of the microbiota in the course of chronic diseases including CKD [136].

### 3.7. Education and Updating of Health Professionals, Patients and Family Members

The actions of education and updating should largely be directed at the patients’ main caregivers, i.e., family members or care assistants who make informed purchases of foods paying particular attention to phosphorus, potassium, and protein content, which are fundamental for dialysis patients. It goes without saying that the use of fresh food is preferable to use of processed foods [137]. Prior to the advent of hemodialysis program treatments, the International Society of Renal Nutrition and Metabolism working group carried out a prospective, interventional study known as the Nutritional Education Program on a total of 160 patients with CKD [138]. It was demonstrated that the actions of those who shop or cook is of fundamental importance in avoiding the purchase of processed foods containing phosphates, potassium, sodium, sulfites, etc., which have preservative, thickening and stabilizing functions [139]. Appropriate nutrition should also provide for an adequate energy intake, in healthy individuals amounting to 31.8 ± 7.0 kcal/kg/day; however, in hemodialysis patients, dietary intake is frequently insufficient, reaching 29.5 ± 6.6 kcal/kg/day [140]. Physical activity in CKD patients should also be included as part of a therapeutic program and be increased [141]. Exercises such as push-ups, pull-ups, crunches, air squats, Pilates, and aerobic endurance exercises designed to increase cardiovascular and respiratory fitness, such as walking or running, are recommended [142].

## 4. Conclusions

A series of tools are currently available for interventions required in cases of malnutrition and protein-energy wasting, achieving satisfactory results both in patients on conservative treatment and in hemodialysis. However, despite use of the above measures, the evolution of PEW is unstoppable in patients having no immediate prospects of transplantation, particularly elderly patients. Regardless of the use of excellent and refined dialytic strategies, unfortunately only a portion of uremic toxins are purified, thus resulting in inflammation, hypercatabolism and failure to avoid the loss of numerous substances that could contribute towards stabilizing the uremia metabolism. The only solution both in terms of expectation and quality of life remains renal transplantation. Nevertheless, a series of therapeutic interventions are available to assist in slowing down or halting the onset of metabolic catabolism, including amino acid substitution which, based on the results of recent studies, ranks amongst the most effective. 

## Figures and Tables

**Table 1:** Diagnostic criteria for malnutrition and protein-energy wasting in CKD patients.

Malnutrition	PEW
**first alternative**	**at least three out of the four listed grouping and at least one test in each one**
BMI < 18.5 kg/m^2^	BMI < 23 kg/m^2^; Unintentional weight loss > 10% over 6 months or > 5% over the last three months; total body fat < 10%
**second alternative**	
Unintentional weight loss > 10% (indefinite time) or > 5% over the last three months	serum Albumin < 3.8 g/dL; serum Prealbumin < 30 mg/dL for dialysis patients or according to residual renal function for patients with CKD stages 2-5; serum cholesterol < 100 mg/dL
BMI < 20 kg/m^2^ if 70 years of age or < 22 kg/m^2^ if > 70 years of age	Dietary PI < 0.8 g/kg/day for at least two months for dialysis patients or < 0.6 g/kg/day for patients with CKD stages 2-5. Unintentional energy intake < 25 kcal/kg/day for at least two months
FFMI < 15% and 17% kg/m^2^ in women and men, respectively	MM 5% reduction over three months or 10% over six months; reduction of creatinine appearance

FFMI: free fat mass index; MM: muscle mass; MAMC: mid-arm circumference MAMC area > 10% in relation to 50th percentile of reference population; PI: protein intake.

**Table 2:** Forecast Losses of Total Amino Acids (TAAs) through hemodialysis according to different timings and regimens.

Session Time Schedule	TAAs Losses/g/year
Thrice weekly, 4 h	800–810
Four-Fold Weekly, 4 h	1000–1100
Long Thrice Weekly Hemodialysis, 8 h	2000–2100
Daily Hemodialysis with time schedule of 2.5–3 h	1000–1200

**Table 3:** Preliminary trial of a group taking AA supplementation compared to a placebo control group [88].

	Control Placebo Group = n.14		Study Supplemented Group = n.15	
	Baseline	3 Months	Baseline	3 Months
Body Weight, Kg	59.1 ± 12.7	58.8 ± 5.8	69.8 + 13.7	68.9 + 13.5 ^a^
BMI, kg/m^2^	25.9 ± 5.8	25.4 ± 5.8	28.6 ± 5.6	28.5 ± 5.5
eKt/V	1.39 ± 0.22	1.38 ± 0.16	1.23 ± 0.26	1.34 ± 0.16
ePCR, g/kg/d	0.9 ± 0.2	0.9 ± 0.2 **	0.9 ± 0.2 ^b^	1.1 ± 0.2 ** ^b^
Phase angle, (°)	4.8 ± 1	4.8 ± 0.7	4.6 ± 0.9	4.9 ± 1
FFM, kg	41.5 ± 6.6	42.1 ± 6.0 *	39.5 ± 6.6 *	38.1 ± 6.3 *
FM, kg	27.9 ± 10.6 *	27.7 ± 11.6 *	22.1 ± 7.8 *	22.6 ± 7.5 *
Albumin, g/dL	3.19 ± 0.16	3.09 ± 0.31 ***	3.08 ± 0.29 ^c^	3.58 ± 0.23 *** ^c^
Total Proteins, g/dL	5.91 ± 0.49	5.95 ± 0.46 *	5.70 ± 0.41 ^c^	6.43 ± 0.73 * ^c^
Hb, g/dL	11.0 ± 0.7	10.6 ± 0.6 ***	10.7 ± 0.9 ^a^	11.7 ± 0.8 *** ^a^
ERI, U/Kg/week/g. Hb	15.2 ± 14.8	14.7 ± 16.8 ^a^	13.1 ± 12.8	12.7 ± 15.5 ^a^
BUN, mg/dL	60.1 ± 13.7	59.5 ± 14.9	60.9 ± 0.8	64.4 ± 0.7
CRP, mg/L	13.6 ± 17.1	11.2 ± 12.2 **	8.7 ± 7.3 ^b^	3.8 ± 3.1 ** ^b^
Tot. Ig, mg/dL	1359 ± 237	1304 ± 222	1249 ± 548	1549 ± 470 ^b^
C3, mg/dL	98.6 ± 27.6	93.8 ± 10	41.5 ± 6.6	97.3 ± 12.8

BMI (body mass index); eKt/V (equilibrated Kt/V); ePCR (equilibrated protein catabolic rate); FFM (free fat mass); FM (fat mass); ERI (erythropoietin resistance index); BUN (blood urea nitrogen); CRP (C reactive protein); Ig (immunoglobulin). a: *p* < 0.05, b: *p* < 0.01, c: *p* < 0.001 vs. baseline; *: *p* < 0.05, **: *p* < 0.01, ***: *p* < 0.001 vs. Control Group.
